# Flux Growth, Crystal Structure, and Chemical Bonding of Yb_2_PdGe_3_, an AlB_2_ Superstructure within the Rare-Earth Series

**DOI:** 10.1021/acs.inorgchem.2c03303

**Published:** 2023-01-20

**Authors:** Riccardo Freccero, Laura C. J. Pereira, Pavlo Solokha, Serena De Negri

**Affiliations:** † Dipartimento di Chimica e Chimica Industriale, Università degli Studi di Genova, Via Dodecaneso 31, I-16146Genova, Italy; ‡ Centro de Ciências e Tecnologias Nucleares, Department of Engenharia e Ciências Nucleares, Instituto Superior Técnico, Universidade Lisboa, Estrada N acional 10, 2695-066Bobadela, Portugal

## Abstract

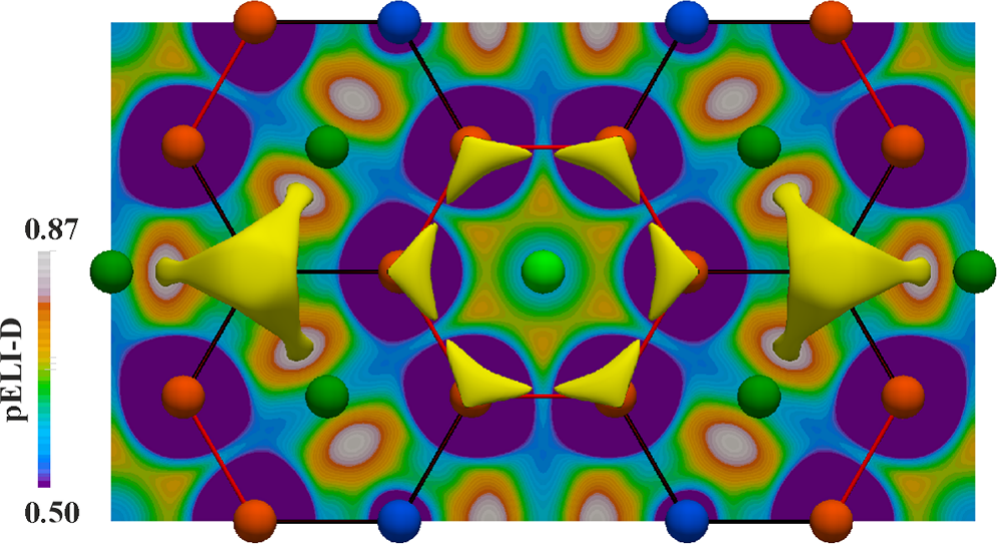

The complete structure revision of the *RE*_2_PdGe_3_ (*RE* = rare-earth metal) series revealed that Yb_2_PdGe_3_ is the only AlB_2_ ordered superstructure. Good-quality single crystals of this compound were successfully grown from molten indium flux, enabling accurate single-crystal investigations. Yb_2_PdGe_3_ crystallizes with the Ce_2_CoSi_3_-type structure in the hexagonal space group *P*6/*mmm* (no. 191) with lattice parameters *a* = 8.468(1) Å and *c* = 4.0747(7) Å. This structure is a four-order derivative of AlB_2_, composed of planar _∞_^2^[PdGe_3_] honeycomb layers spaced by Yb species, located at the center of Ge_6_ and Ge_4_Pd_2_ hexagons. A superconducting transition is observed below the critical temperature of 4 K. A divalent state of Yb is deduced from magnetic susceptibility measurements below room temperature, which indicate an almost nonmagnetic behavior. A charge transfer from Yb to Pd and Ge was evidenced by the Quantum Theory of Atoms in Molecules (QTAIM) effective charges; polar four-atomic Ge–Pd/Yb and two-atomic Pd–Yb bonds were observed from the ELI-D (electron localizability indicator), partial ELI-D, and ELI-D/QTAIM intersections. The bonding interactions between Ge atoms within regular Ge_6_ hexagons are found to be intermediate between single bonds, as in elemental Ge, and higher-order bonds in the hypothetic Ge_6_H_6_ and Ge_6_^6–^ aromatic molecules.

## Abstract 2

The Yb_2_PdGe_3_ intermetallic was found to be the only AlB_2_ ordered superstructure within the *RE*_2_PdGe_3_ (*RE* = rare-earth metal) series. Single crystals of such phase were successfully grown in indium flux. Yb_2_PdGe_3_ is a type II superconductor composed of two-dimensional _∞_^2^[PdGe_3_] honeycomb anionic layers spaced by Yb cations. The Ge–Ge interactions are analyzed based on a comparative analysis with the Ge_6_H_6_ and Ge_6_^6–^ molecules.

## 1 Introduction

Ternary rare-earth tetrelides *RE*–*T*–*Tt* (*RE* = rare-earth metal; *T* = transition metal; *Tt* = tetrel element) have been heavily studied due to their intriguing structure peculiarities, unconventional physical properties, and unprecedented bonding scenarios.^1−10^ Among them, intermetallics crystallizing with a disordered AlB_2_-type structure or with one of its ordered derivatives^11^ have attracted particular attention since the discovery of superconductivity with *T*_c_ ∼ 39 K in MgB_2_,^12^ featuring similar honeycomb-like layers. Unfortunately, investigations of physical properties of these compounds were frequently not accompanied by accurate and in-depth structural analyses, which are indeed crucial to enable a correct interpretation of the observed phenomena. This is the case for compounds with 33.3 atom % *RE*; 16.7 atom % Pd; and 50.0 atom % *Tt* nominal composition, corresponding to the 2:1:3 stoichiometry.^13^ As shown in Table 1, both silicides and germanides were found to exist with almost all of the *RE*s.^14^

**Table 1:**
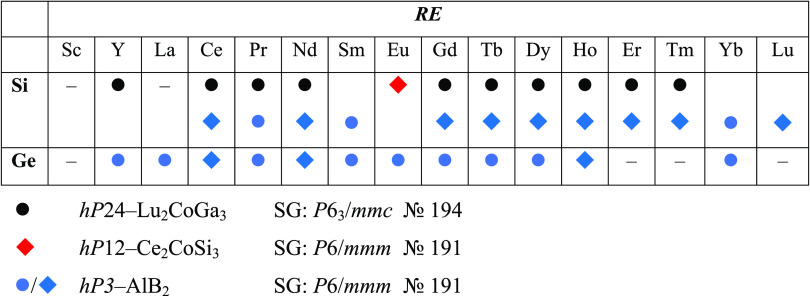
Crystal Structures Reported in the Literature for *RE*–Pd–{Si, Ge} Phases of 2:1:3 Composition^a^

a ● indicates that only cell parameters and the structure type were assigned and ◆ indicates a complete structure determination.

Most of the silicides were first reported as *hP*24-Lu_2_CoGa_3_, an ordered superstructure of AlB_2_,^11^ and subsequently revised in some cases as disordered, i.e., *hP*3-AlB_2_, based on more accurate structural investigations. The Eu-containing compound is the only one crystallizing with the *hP*12-Ce_2_CoSi_3_ structure, which is an AlB_2_ derivative as well.^11^ Moving to Ge, no superstructures have been reported;^15^ nevertheless, a complete structure determination was performed only in a few cases, i.e., with Ce, Nd, and Ho. Although many of such phases have been described as disordered and assigned to the AlB_2_ type, they were often referred to with the misleading *RE*_2_Pd*Tt*_3_ formula, instead of *RE*(Pd_*x*_Ge_1–*x*_)_2_ (with *x* = 0.25). Additionally, it is worth mentioning that the formation of an ordered Lu_2_CoGa_3_ or Ce_2_CoSi_3_ superstructure would lead to symmetrically inequivalent *RE* sites, which must be considered to correctly interpret the measured magnetic properties. In fact, both ferro^16−18^ and antiferromagnetic^13,17^ order were detected in several cases. Interestingly, “Y_2_PdGe_3_” is the only representative displaying a superconducting behavior below ∼3 K.^16,19^ Given this unique feature and the less accurate crystallographic data reported for the germanides with respect to the silicides, a complete structural reinvestigation along the whole *RE* series was performed for the “*RE*_2_PdGe_3_” phases, paying particular attention to the possible formation of ordered structures. As the main focus of this paper, we report on the successful synthesis of the novel Yb_2_PdGe_3_ intermetallic, prepared both from metal flux (indium) and by direct synthesis. Its crystal structure solution and group–subgroup relations with AlB_2_ are presented together with magnetic properties and chemical bonding analysis. In fact, the involved elements together with the structural features based on Ge–Ge interactions suggest approaching the chemical bonding, applying the Zintl–Klemm concept. To explain the deviations from this ideal model, it is necessary to investigate the alternative ways through which the system compensates for insufficient charge transfer and electron deficiency. For instance, in molecular chemistry, this is achieved by the formation of multiple bonds comprising π-interactions as in aromatic molecules. The similarity between the honeycomb-like layers in the compounds of interest and the widespread hexagonal aromatic cycles, together with previous studies on intermetallic π-systems,^20−22^ inspired a comparative chemical bonding analysis between Yb_2_PdGe_3_ and ad hoc simulated molecular analogues. This contribution highlights the importance of a more general perspective in studying chemical bonding, overcoming the classic boundaries between solid-state and molecular chemistry.

## 2 Experimental Section

### 2.1 Synthetic Procedures

Samples of nominal composition 33.3 atom % *RE*; 16.7 atom % Pd; and 50.0 atom % Ge (*RE* = Y, La–Nd, Sm, Gd–Er, Yb) were synthesized to check for the existence of the ordered *RE*_2_PdGe_3_ phase. The starting materials were rods of the rare-earth metals (supplied by NewMet Ltd., Waltham Abbey, U.K.) with a freshly cleaned surface, palladium foils, and germanium chunks (supplied by MaTecK, Jülich, Germany), all with a nominal purity > 99.9 mass %. Ingots of about 0.8 g were obtained by melting stoichiometric amounts of the pristine elements.

Samples with *RE* = Y, La–Nd, Gd, Tb, Er were prepared by arc melting on a water-cooled copper heart with a tungsten electrode under ∼1 bar of Ar gas. The obtained alloys were flipped and arc-melted multiple times, ensuring their homogeneity. Weight losses were always lower than 1%.

Samples with *RE* = Sm, Dy, Ho, Yb were prepared by induction melting of the elements enclosed in arc-welded Ta crucibles to avoid element losses. The melting procedure was performed under a continuous argon flow, to prevent the high-temperature oxidation of the crucible, and repeated several times to guarantee homogeneity. These samples were characterized both in the as-cast state and after annealing at 800 °C for 3 weeks.

To obtain single-phase samples of Yb_2_PdGe_3_ necessary for physical properties measurements, stoichiometric amounts of the constituents were inserted in an arc-welded Ta crucible and then closed in an evacuated quartz ampoule to avoid oxidation. Subsequently, the ampoule was hung in a resistance furnace and submitted to the following thermal cycle while rotating at a speed of 100 rpm: heating (10 °C min^–1^) up to 750 °C; heating (1 °C min^–1^) up to 950 °C; cooling (∼0.2 °C min^–1^) down to 800 °C. Then, the rotation was disabled, the sample kept at 800 °C for 1 week, and finally water quenched. The resulting alloy of metallic luster was ground to fine powders in an agate mortar and pressed into a pellet. The pellet was enclosed in an arc-sealed Ta crucible, put in an evacuated quartz phial, and annealed at 800 °C for 1 month prior to quenching in water.

Single crystals of Yb_2_PdGe_3_ suitable for X-ray diffraction analysis were grown from an indium flux. For this purpose, stoichiometric amounts of Yb, Pd, and Ge giving the Yb_33.3_Pd_16.7_Ge_50.0_ nominal composition were placed in an arc-sealed Ta crucible with 1:45 molar excess of In (chunk, 99.99%, supplied by NewMet Ltd., Waltham Abbey, U.K.). The total mass was of about 3 g. Thus, the Ta crucible, closed in an evacuated quartz ampoule, was hung in a resistance furnace, heated up to 750 °C in about 1 h, and kept at that temperature for 1 day. Then, it was cooled to room temperature in 24 h. During the whole treatment, a continuous rotation at 100 rpm was applied.

Aiming at separating the single crystals of Yb_2_PdGe_3_ from the In flux, pieces of the obtained ingot were laid down on a glass wool filter and sealed in a quartz tube. The specimen was then preheated at 300 °C in a resistance furnace and centrifugated at a speed of 600 rpm for about 1 min. This procedure was repeated several times, enabling to obtain shiny gray crystals of Yb_2_PdGe_3_. Residual indium deposited on the crystal surfaces was selectively oxidized by immersion and sonication of the crystals in glacial acetic acid for about 2 h.

### 2.2 Scanning Electron Microscopy and Elemental Analysis

Metallographic analysis was performed on each sample. Small pieces were embedded in a conductive carbon-containing phenolic resin by means of an automatic hot compression mounting press Opal 410 (ATM GmbH, Germany) and submitted to a multistep grinding (SiC papers from 600 to 1200 mesh) and polishing (from 6 to 1 μm diamond pastes) procedure with the aid of an automatic polishing machine Saphir 520 (ATM GmbH, Germany). After each polishing step, sample surfaces were cleaned for a few minutes in an ultrasonic bath using petroleum ether. Several Yb_2_PdGe_3_ single crystals separated from the In flux were placed on a conductive carbon resin and analyzed as such. Microstructure examination as well as semiquantitative elemental analysis of the observed phases were performed using a scanning electron microscope (SEM) Zeiss Evo 40 (Carl Zeiss SMT Ltd., Cambridge, England), equipped with an energy dispersive X-ray (EDX) spectroscope from Oxford Instruments (INCA X-ACT). The calibration was effectuated on a cobalt standard.

### 2.3 X-ray Diffraction (XRD) Experiments

**Figure 1:**
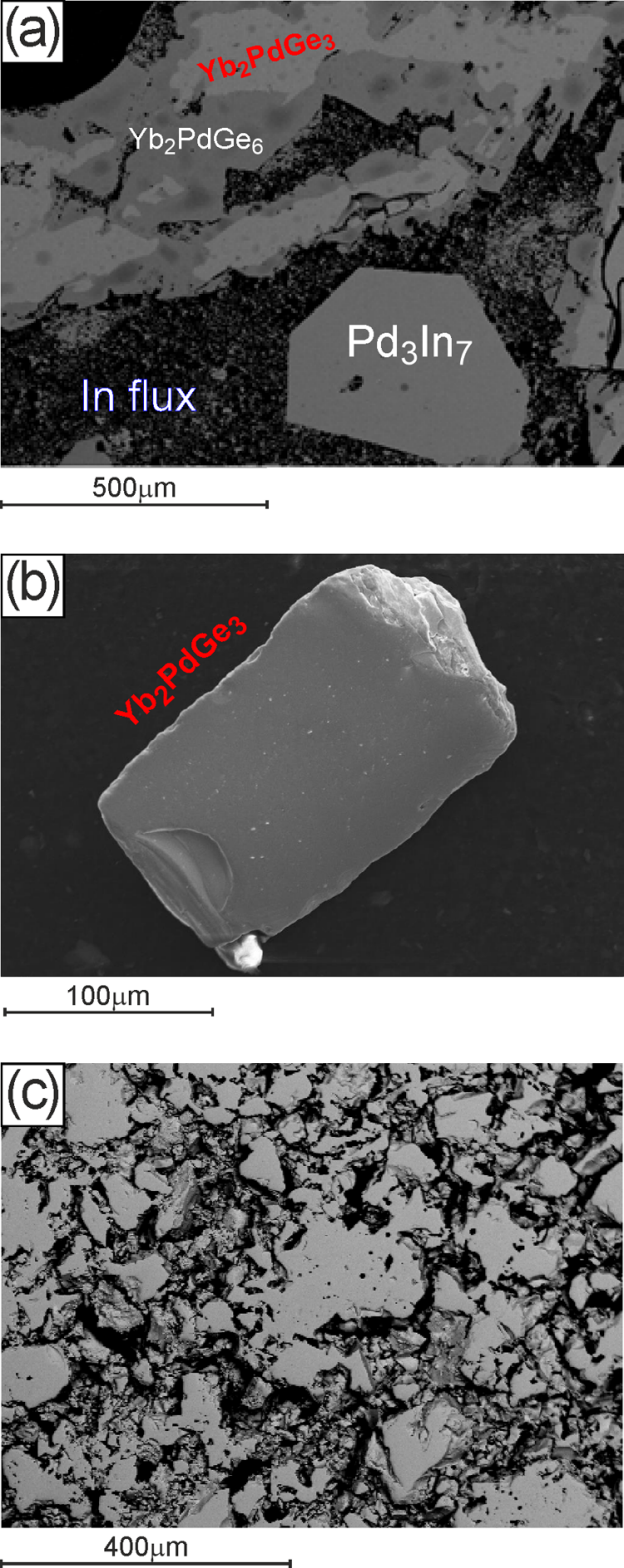
SEM micrographs of Yb–Pd–Ge samples: (a) synthesized by flux method, prior In removal (bulk)-BSE mode; (b) synthesized by flux method, after In removal (single crystal)-SE mode; and (c) obtained by direct synthesis followed by sintering at 800 °C-BSE mode.

Single crystals of Yb_2_PdGe_3_ with metallic luster were extracted both from the mechanically fragmented sample prepared by direct synthesis and from the metal flux medium (In) after suitable centrifugation and selective oxidation. Due to their quality (see Figure 1), the flux-separated crystals were measured by single-crystal X-ray diffraction. A complete data set was obtained in a routine fashion at ambient conditions on a three-circle Bruker Kappa APEXII CCD area-detector diffractometer equipped by the graphite monochromatized Mo Kα (λ = 0.71073 Å) radiation, operating in ω-scan mode. Crystals were glued on glass fibers, mounted on a goniometric head, and placed in the goniostat. Intensity data were collected over the reciprocal space up to ∼30° in θ (achieving a ∼0.7 Å resolution) with exposures of 20 s per frame.

Data collection was performed, and the unit cell was initially refined using APEX4 [v2021.10-0].^23^ Successively, data were reduced using SAINT [v8.30A]^24^ and XPREP [v2014/2].^25^ Lorentz, polarization, and absorption effects were corrected using SADABS [v2016/2].^26^ The structure was solved and refined with the aid of the programs JANA2006^27^ and SHELXL-2019/1.^28^

The corresponding CIF file, available in the Supporting Information, has been deposited at the Cambridge Database with the depository number CSD-2230957. Selected crystallographic data and structure refinement parameters for the studied single crystal are listed in Table 2. Details on the structure solution are discussed in Section 3.2.

**Table 2:** Crystallographic Data for Yb_2_PdGe_3_

empirical formula	Yb_2_PdGe_3_
EDXS data	Yb_34.2_Pd_17.6_Ge_48.2_
space group, *Z*	*P*6/*mmm*, 2
Pearson’s symbol/prototype	*hP*12-Ce_2_CoSi_3_
*M*_w_[g/mol]	670.42
*a* [Å]	8.468(1)
*c* [Å]	4.0747(7)
*V* [Å^3^]	253.06(7)
calc. density [g/cm^3^]	8.796
abs coeff (μ), mm^–1^	57.5
unique reflections	204
reflections *I* > 2σ(*I*)	200
*R*_sigma_	0.0143
data/parameters	204/13
GOF on *F*^2^ (S)	1.46
*R* indices [*I* > 2σ(*I*)]	*R*_1_ = 0.0188; w*R*_2_ = 0.0782
*R* indices [all data]	*R*_1_ = 0.0193; w*R*_2_ = 0.0785
Δρ_fin_(max/min), [e/Å^3^]	1.74/–1.67

X-ray powder diffraction (XRPD) measurements were performed on all samples using a Philips X’Pert MPD vertical diffractometer (Cu Kα radiation, λ = 1.5406 Å, graphite crystal monochromator, PIXcel^1D^ detector). Rietveld refinement was conducted using the Fullprof^29^ software on the powder pattern of the sample submitted to physical properties measurements.

### 2.4 Physical Properties Measurements

Magnetic measurements were carried out using a 7 T Squid magnetometer (S700 X from Cryogenics, Ltd.) on a polycrystalline sample with an approximate mass of 10–20 mg inside a transparent capsule of 5 mm diameter. Diamagnetic signals from the gelatine capsule and straw were corrected; ZFC (zero-field cooling) and FC (field cooling) magnetic susceptibility curves were taken at different DC magnetic fields, (2.5, 5, and 10 mT) in the temperature range 1.6–300 K. Isothermal magnetization curves and hysteresis loops up to 5 T at selected temperatures were also obtained.

### 2.5 Computational Details

The electronic structure of Yb_2_PdGe_3_ was calculated by means of the all-electron full-potential local-orbital FPLO code^30,31^ using the experimentally determined structure. The local spin density approximation (LSDA) to the density functional theory (DFT) as parametrized by Perdew and Wang (PW)^32^ were employed to account for exchange and correlation. Relativistic effects were treated at the scalar-relativistic level. Moreover, the LSDA+*U* method was applied due to the localized nature of the Yb 4*f* states. The on-site Coulomb repulsion parameter *U* was set to the characteristic FPLO value of 8 eV.^33,34^ The atomic limit (AL) method was selected as the double-count correction scheme. The Brillouin zone was sampled with a (4 4 8) *k*-point mesh. Position-space chemical bonding analysis was performed by combining topological analysis of the electron density (ED) and the electron localizability indicator, in its ELI-D^35−37^ representation, using the software DGrid.^38,39^ The two scalar fields were both calculated in an equidistant grid of about 0.05 Bohr using an implemented module within the FPLO code.^40^ The ED was analyzed within the framework of the Bader’s Quantum Theory of Atoms In Molecules (QTAIM).^41^ For this purpose, the crystal space was partitioned into nonoverlapping and space-filling regions, the atomic basins, based on the gradient vector field of the ED. Its integration within each QTAIM atom gives its average electronic population that is subtracted from the atomic number providing the atomic effective charges (*Q*^eff^). The application of the same procedure to the ELI-D separates the crystal space into core and valence basins, giving access to bonding interactions among the constituents. Molecular calculations for hexagermanbenzene, Ge_6_H_6_, and Ge_6_^6–^ were effectuated with the all-electron FHI-aims software. Atomic coordinates were optimized starting from planar geometries using the LDA/PW, GGA/PBE,^42^ and B3LYP^43^ exchange and correlation functionals. For both Ge and H, the predefined default “tight” basis sets were chosen and scalar-relativistic effects for all electrons were taken into account within the zero-order regular approximation (ZORA). The ED, ELI-D, and partial ELI-D (pELI)^36,44^ were evaluated using the program DGrid^39^ on the basis of the obtained wave functions. Scalar fields and the related basins for both Yb_2_PdGe_3_ and the molecules were visualized with the aid of the ParaView^45,46^ application.

## 3 Results and Discussion

### 3.1 Results of SEM/EDXS/XRPD Characterization

Characterization of the *RE*_33.3_Pd_16.7_Ge_50.0_ samples (*RE* = Y, La–Nd, Sm, Gd–Er) confirmed the literature data on the existence of the *RE*(Pd*_x_*Ge_1–*x*_)_2_ phases with an average composition close to the 2:1:3 stoichiometry and an AlB_2_-like crystal structure, where Pd and Ge share the 2*d* crystallographic site; phases comprising La and Ce turned out to be tetragonal (see Table S1). The Er-containing member of this series is here reported for the first time. As an example, the X-ray powder patterns of *RE*(Pd*_x_*Ge_1–*x*_)_2_ phases (*RE* = Ce, Pr, Tb) are shown in Figure S1.

However, the Yb-containing samples, prepared both by metal flux and direct synthesis followed by sintering, showed the presence of the Yb_2_PdGe_3_ ternary phase (see Table 3) with an ordered superstructure (see Section 3.2 for crystal structure details).

**Table 3:** Results of SEM/EDXS/XRPD Characterization for Yb_33.3_Pd_16.7_Ge_50.0_ Samples Prepared by Metal Flux (#1) and Direct Synthesis in Resistance Furnace (#2)

		composition by (EDXS) [atom %]					lattice parameters [Å]		
sample code synthesis	phases	Yb	Pd	Ge	In	Pearson symbol prototype	*a*	*b*	*c*
#1	Yb_2_PdGe_3_	34.2(3)	17.6(4)	48.2(3)		*hP*12-Ce_2_CoSi_3_	8.474(2)		4.073(2)
	Yb_2_PdGe_6_	23.0(2)	12.1(2)	64.9(1)		*oS*72-Ce_2_(Ga_0.1_Ge_0.7_)_9_	8.150(2)	7.990(1)	21.847(5)
	Pd_3_In_7_		31.4(3)		68.6(3)	*cI*40-Ru_3_Sn_7_	9.4275(6)		
	Ge			100.0		*cF*8-C	5.654		
#2	Yb_2_PdGe_3_	34.6(6)	16.9(7)	48.5(7)		*hP*12-Ce_2_CoSi_3_	8.4629(4)		4.0733(4)

Indium flux turned out to be reactive: in sample #1, Pd_3_In_7_, Yb_2_PdGe_6_, recrystallized Ge and the new Yb_2_PdGe_3_ were detected both by SEM-EDXS (Figure 1a) and X-ray powder diffraction obtained after the flux separation (see Figure S2). The peaks associated to tetragonal In were not revealed in the X-ray powder pattern. Good-quality Yb_2_PdGe_3_ crystals, showing a plate-like morphology, were detected with SEM/EDXS (Figure 1b) with no traces of In impurities. From this sample, a single crystal was selected for X-ray analysis and structure solution.

Sample #2 was revealed to be Yb_2_PdGe_3_ single phase (see Figure 1c) with the same crystal structure as in the In-flux synthesized sample.

### 3.2 Crystal Structure of Yb_2_PdGe_3_ as the AlB_2_ Superstructure

A Yb–Pd–Ge ternary compound of composition ∼33.3 atom % Yb; ∼16.7 atom % Pd; and 50 atom % Ge was reported in the literature^47^ as AlB_2_-like (*a* = 4.2276(3) Å, *c* = 4.0686(6) Å), as the representatives with other rare-earth components.

The analysis of the collected data highlights that the strongest diffraction peaks are compatible with the AlB_2_-type pattern; however, a regular distribution of weak superreflections cannot be neglected; including all of these in the indexation, a hexagonal unit cell with *a* ∼ 8.47 Å, *c* ∼ 4.07 Å is obtained, that is four times bigger than that of the AlB_2_ type.

**Figure 2:**
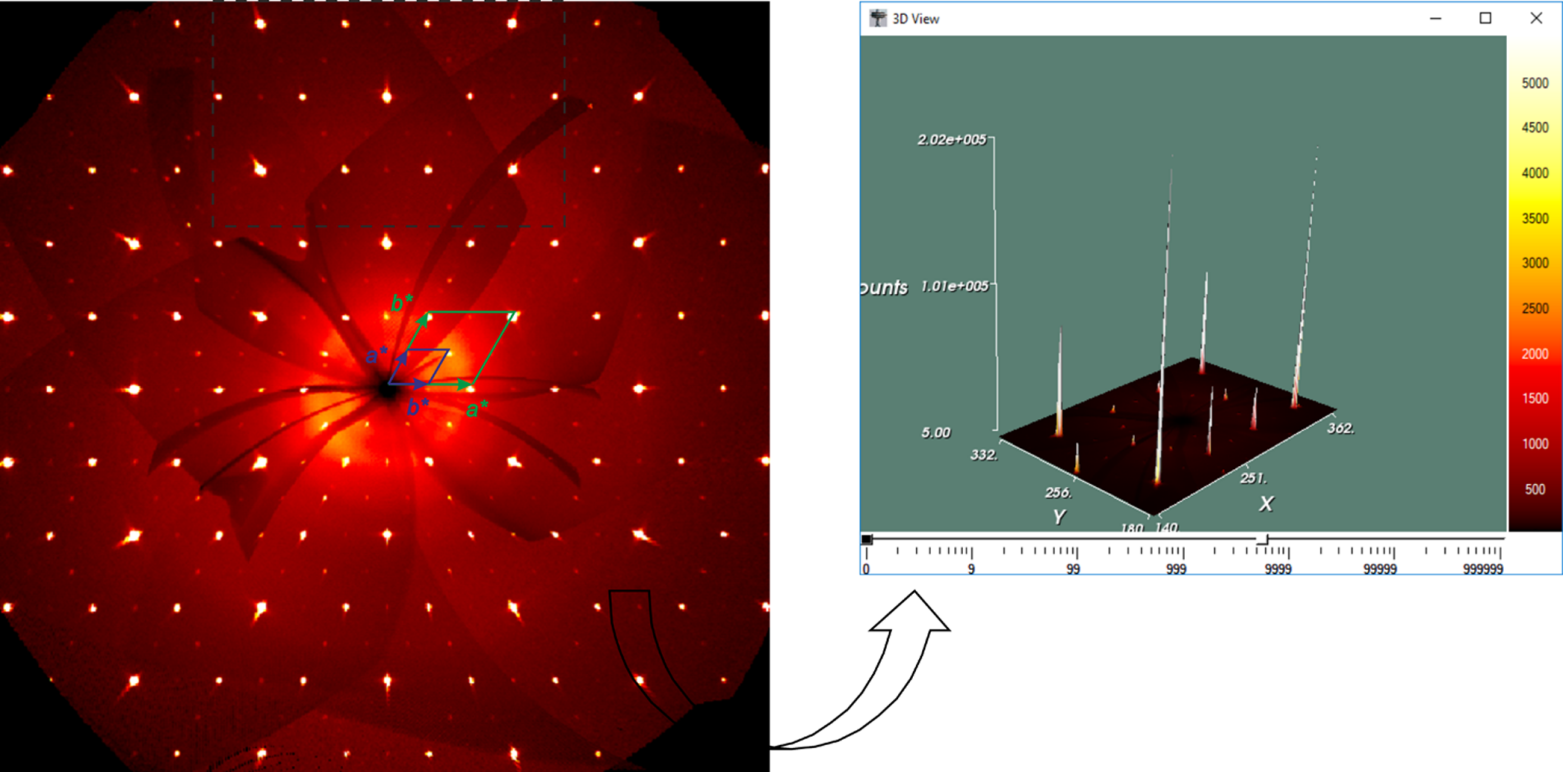
Intensity profiles for *hk*0 zone, with the unit cells of AlB_2_ parent type (green) and of the Yb_2_PdGe_3_ superstructure (blue). The presence of weak superreflections is well visible in the 3D view (on the right).

In the reconstructed *hk*0 precession image, the reciprocal space relation between parent and derivative unit cells is evidenced (Figure 2, left); the intensity difference between the main and superreflections is well visible from the 3D plot (Figure 2, right).

The analysis of the systematic absences suggests a primitive lattice centering and numerous possible space groups (*P*6/*mmm*, *P*622, *P*6*mm*, *P*-6, *P*6, etc.). Moreover, the |*E*^2^ – 1| criterion was ∼1.4, being noticeably far from the ideal value of 1 (centrosymmetric space group). These observations can be reasonably explained by the elevated number of weak superreflections in the data set. A chemically reasonable structure model was found in the *P*6/*mmm* space group. Further structure refinements were carried out by full-matrix least-squares methods on |*F*^2^| using the SHELXL program package.^28^ The site occupancy factors of all species were checked for deficiency, in separate cycles of refinement, obtaining values very close to unity. The final model was additionally checked with PLATON,^48^ indicating no missing symmetry elements. At this point, neither deficiency nor the statistical mixture were considered, and the stoichiometric Yb_2_PdGe_3_ (*hP*12-Ce_2_CoSi_3_) model was further anisotropically refined, giving acceptable residuals and a flat difference Fourier map. Selected crystallographic data are listed in Table 4.

**Table 4:** Atomic Coordinates and Equivalent Isotropic Displacement Parameters for the Investigated Yb_2_PdGe_3_ Single Crystal

atom	site	*x*/*a*	*y*/*b*	*z*/*c*	*U*_eq_ (Å^2^)
Yb1	3*f*	1/2	0	0	0.0107(3)
Yb2	1*a*	0	0	0	0.0090(3)
Pd	2*d*	1/3	2/3	1/2	0.0103(3)
Ge	6*m*	0.16616(2)	0.33232(3)	1/2	0.0108(3)

**Figure 3:**
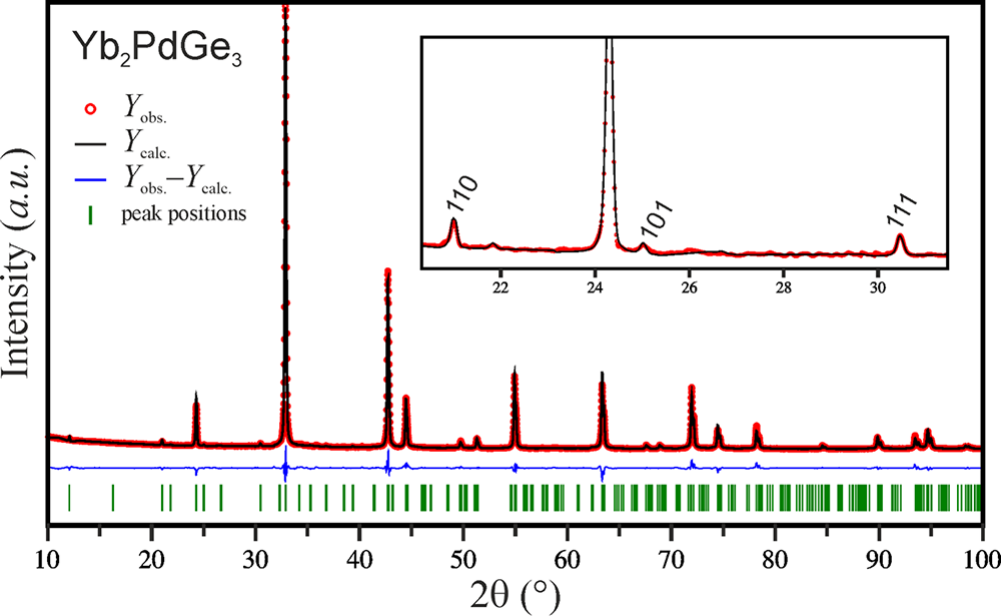
Observed (red circles), calculated (black line), and difference (bottom blue line) X-ray powder diffraction patterns for Yb_2_PdGe_3_. Indexed superreflections are visible in the inset.

The structural model was confirmed by X-ray powder diffraction analysis on the Yb_2_PdGe_3_ sample prepared by direct synthesis followed by sintering. Results of Rietveld refinement on this sample are visualized in Figure 3. Least-squares refinement cycles converged to *R*_B_ = 0.0512, *R*_F_ = 0.109, and χ^2^ = 3.93, confirming that the diffraction pattern calculated based on the established structural model of Yb_2_PdGe_3_ is in good agreement with the experimental data. The refined lattice parameters and atomic positions are in good agreement with single-crystal data.

**Figure 4:**
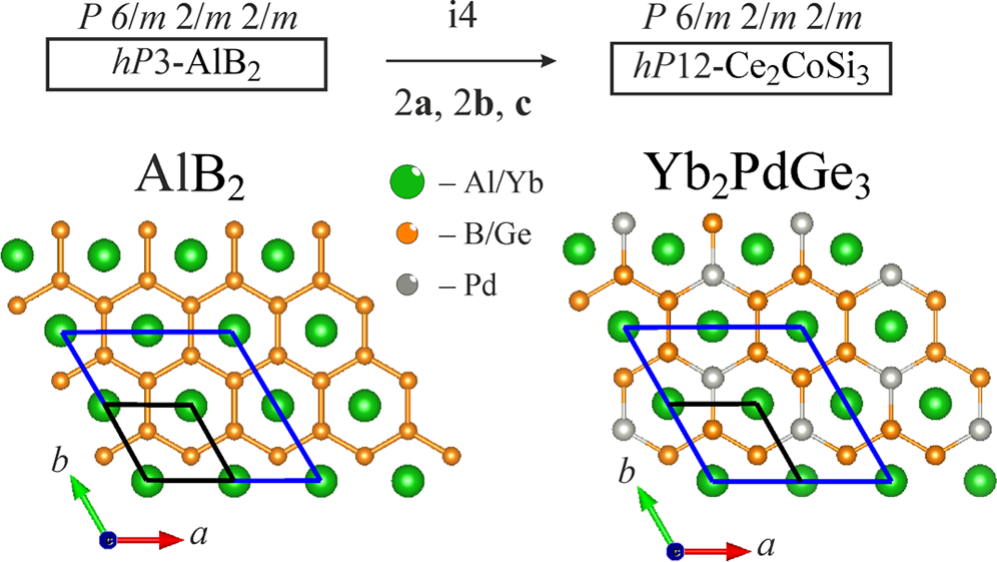
Bärnighausen symmetry reduction step relating the AlB_2_ aristotype and its Yb_2_PdGe_3_ derivative. The graphite-like layers composed of B_6_ (AlB_2_) and Ge_6_/Ge_4_Pd_2_ (Yb_2_PdGe_3_) are evidenced. The unit cells shown in black and blue highlight the metric relations between AlB_2_ and Yb_2_PdGe_3_, respectively.

The relationship between AlB_2_ (aristotype) and Yb_2_PdGe_3_ (four-order superstructure) is conveniently represented in terms of a group–subgroup relation already described in ref.^11^ Starting from AlB_2_, an *isomorphic* transition of index 4 (*i*4) yields to the Yb_2_PdGe_3_ structure, with doubled *a* and *b* axes (see Figure 4). As a consequence, Yb atoms occupy two different positions (1*a* and 3*f*), located in the correspondence to the centers of Ge_6_ and Ge_4_Pd_2_ hexagons, respectively, when viewed along the *c* direction. In each Ge_4_Pd_2_ hexagon, the Pd atoms are placed in *para* positions. Based on Ge–Ge (2.44 Å) and Ge–Pd (2.45 Å) interatomic distances, it is reasonable to interpret their planar layers as covalently bonded. Thus, the study of chemical bonding is of great interest in the framework of the chemistry of inorganic “graphene”.^49,50^

**Figure 5:**
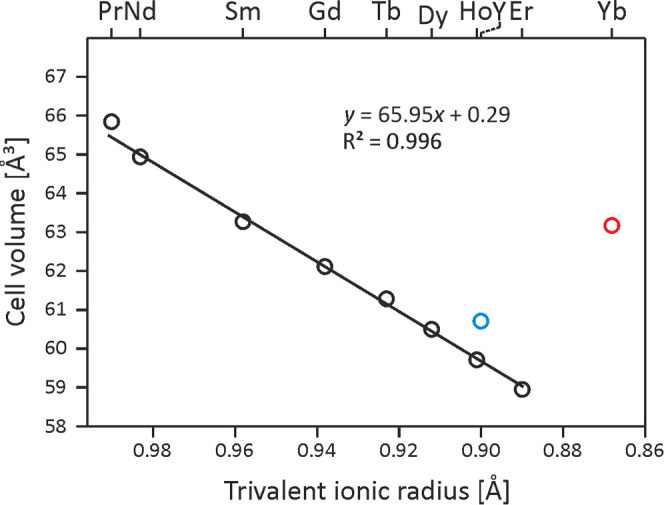
Cell volumes of *RE*(Pd*_x_*Ge_1–*x*_)_2_ and Yb_2_PdGe_3_ (red circle) compounds as a function of the *RE*^3+^ ionic radius. The blue circle indicates the datum for *RE* = Y.

The cell volumes of studied phases as a function of the *RE*^3+^ radius are plotted in Figure 5. A four times smaller cell volume (*V*_cell_/4) was considered for the Yb_2_PdGe_3_ superstructure. The general trend is linear, being in line with the lanthanide contraction (the Y representative was not considered for linear regression). However, the datum for ytterbium is out of trend, suggesting a divalent or intermediated/mixed state for this species and motivating measurements of physical properties. Finally, it is worth noting that the lattice constants found for the *RE*(Pd_*x*_Ge_1–*x*_)_2_ phases are in good agreement with those already published.^14^

### 3.3 Magnetic Properties of Yb_2_PdGe_3_

**Figure 6:**
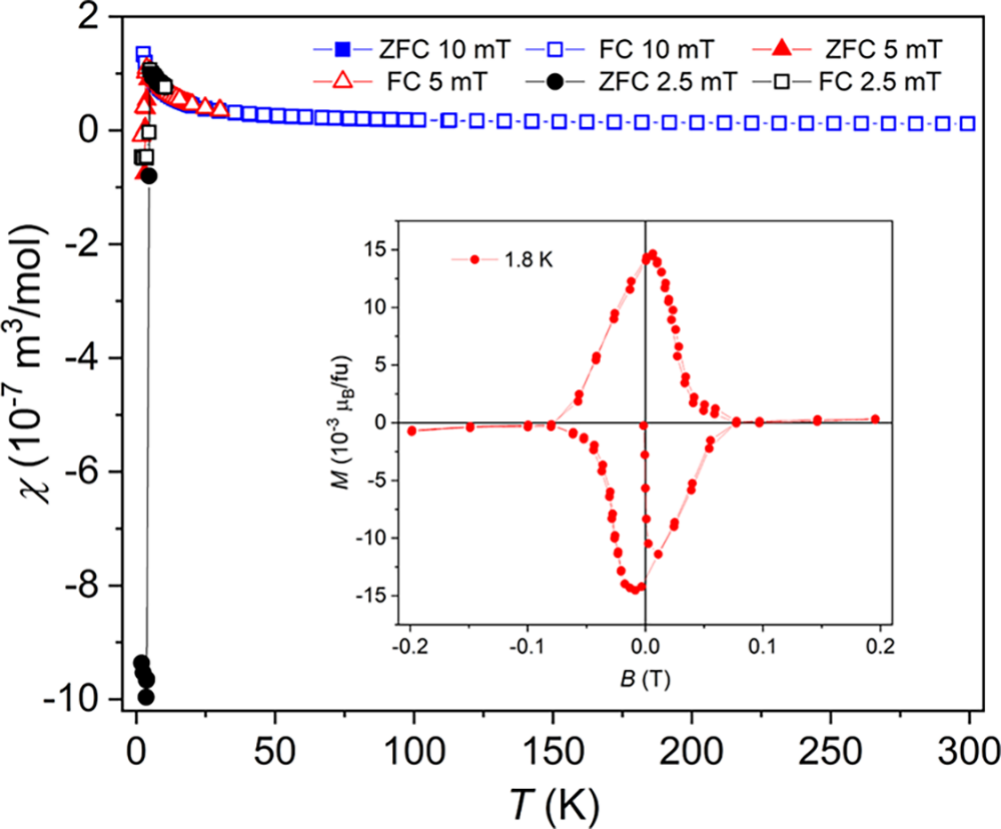
Temperature dependence of the magnetic susceptibility of Yb_2_PdGe_3_ as ZFC/FC cycles taken at 2.5, 5.0, and 10 mT. Inset corresponds to the field dependence of the magnetization below *T*_C_, at 1.8 K.

The temperature dependence of magnetic susceptibility for Yb_2_PdGe_3_ is depicted in Figure 6 for different low-DC fields. Both zero-field-cooled (ZFC) and field-cooled (FC) warming cycles were applied revealing a weak Pauli paramagnetism down to ∼50 K. The residual susceptibility obtained from a linear approximation corresponds to χ_0_ = 1.5 × 10^–8^ m^3^/mol. The upturn in χ(T) below 50 K is probably due to a minor paramagnetic impurity (i.e., invisible in the powder XRD and EDXS). Below 4.5 K, a strong diamagnetic signal appears due to a transition of Yb_2_PdGe_3_ into a superconducting state with a critical temperature, *T*_C_ = 4 K. The appearance of a magnetic hysteresis (Figure 6 inset) confirms the studied germanide to be a superconductor of type II. The superconducting volume fraction is 94% at 2.5 mT and 7.6% at 5.0 mT (see Figure S3). The hysteresis closes at a second critical field *B*_c2_ ≈ 80 mT at 1.8 K. Up to now, in the studied family of compounds, this behavior has only been reported for the Y representative, where a superconducting transition below 3 K was found.^16,19^ A more precise estimation of the superconducting parameters will become an object of further study.

### 3.4 Electronic Structure and Chemical Bonding

Chemical bonding for intermetallic compounds containing *p*-block elements is generally assessed by applying the Zintl–Klemm approach together with a careful crystal-chemical analysis of the polyanionic fragments.

Interatomic Ge–Ge distances (*d*_Ge–Ge_) equal to 2.44 Å leave no doubt about the covalent nature of such interactions. As a first approximation, if compared with *d*_Ge–Ge_ in cubic Ge (2.45 Å),^14^ they may be interpreted as single bonds, leading to the following ionic formula with two homopolar bonds per Ge atom: (Yb^2+^)_2_(Pd^2+^)[(2*b*)Ge^2–^]_3_. At the same time, the presence of planar, regular Ge_6_ hexagons with a 6/*mmm* (*D*_6h_) point symmetry hints toward a Hückel-like arene, formally bearing a −6 charge. In this case, the resulting ionic formula is (Yb^2+^)_4_(Pd^–^)_2_(Ge_6_^6–^). A deeper analysis of interatomic distances for germanides evidences that, although both hypotheses are in line with the 8 – *N* rule, an intermediate bonding scenario might be expected. In fact, Ge–Ge single bonds in Zintl-like compounds are generally expected to be longer than 2.45 Å; this effect is often roughly explained, invoking the Coulombic repulsion among negatively charged Ge species.^51^ The double-bond length in molecular digermenes was reported to range from 2.20 to 2.50 Å^52^ depending on the substituents, being even longer in a few cases. A [Ge_2_]^4–^ Zintl dumbbell, with *d*_Ge–Ge_ = 2.39 Å, was reported by Scherf et al.^51^ within the Li_3_NaGe_2_ phase and described as a solid-state equivalent of O_2_. It is worth mentioning another AlB_2_ derivative, Ba_2_LiGe_3_,^53^ that possesses Ge_6_ rings with *d*_Ge–Ge_ of 2.51 and 2.52 Å. This elongation with respect to the title compound probably stems from the enhanced charge transfer from the metal species, resulting in Ge_6_^10–^ anions, which were described as arene-like π-systems.^53^ At this point, a comparison with the simulated aromatic, Ge_6_H_6_, and Ge_6_^6–^ molecular species could give additional insights. In fact, the shortest *d*_Ge–Ge_ in Yb_2_PdGe_3_ is intermediate between 2.3 Å (Ge_6_H_6_) and 2.7 Å (Ge_6_^6–^) (see Table S2 for further details), prompting an intermediate bonding scenario that may be ascribed to additional Ge–Pd/Yb interactions.

**Figure 7:**
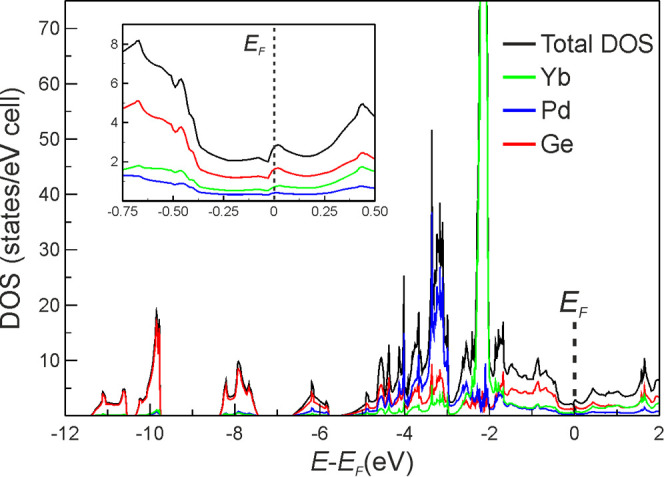
Calculated total and species-projected electronic density of states (DOS/pDOS) for Yb_2_PdGe_3_. The inset displays details around the Fermi level, which is indicated by a dotted line.

This concise survey on interatomic distances shows that a much more complicated bonding scenario than the Zintl one is realized, fostering deeper investigations based on DFT electronic structure calculations. For this purpose, total and species-projected densities of states (DOS and pDOS) were calculated (see Figure 7; the band structure together with the Brillouin zone is shown in Figure S4).

The presence of a pseudogap at the *E*_F_ (see the inset in Figure 7) implies that Yb_2_PdGe_3_ has a metallic behavior. The DOS may be separated into two regions: one below −5.71 eV, which is mainly contributed by the Ge 4*s* states, and the other above, up to *E*_F_. The latter is built up by the Ge 4*p* states that energetically overlap with the Pd and Yb ones, suggesting the formation of polar Ge–Pd/Yb bonds. The active participation of the 4*d* states of Pd into chemical bonding is indicated by their width of about 1 eV. Moreover, the *d* state location far from the *E*_F_, between −5 and −4 eV, hints toward a Pd anionic behavior. The narrow peak at about −2.3 eV is due to the localized and fully occupied 4*f* states of both Yb1 and Yb2 species. If the Coulomb parameter *U* is not employed, the 4*f* states are practically located at *E*_F_ and display a slight increase of the band width (see Figure S5 for the DOS and pDOS obtained from the LDA calculation). The goodness of the selected *U* parameter is also evident when the (p)DOS curves of Yb_2_PdGe_3_ are compared with those of Ca_2_PdGe_3_, calculated using, for consistency, the same computational setup (see Figure S5 to the right). Finally, it is worth noting that although the LSDA + *U* calculations were started with nonzero spin magnetic moments, the self-consistent solution was a nonmagnetic one. These findings support the nonmagnetic nature of the ground state and are consistent with the magnetization data reported in the previous paragraph, revealing a divalent state for Yb species.

**Figure 8:**
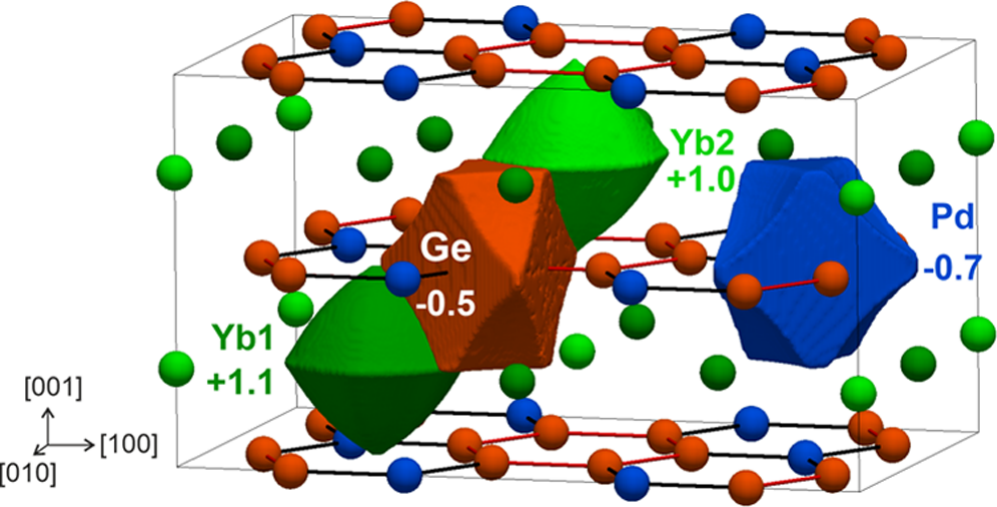
Shapes and effective charges of the QTAIM atomic basins for Yb_2_PdGe_3_. To enable a clearer view, an ortho-hexagonal cell was employed.

To get more insights on chemical bonding, quantum-chemical techniques in position space were selected. The effective QTAIM charges are show in Figure 8. Yb species bear very similar positive charges, i.e., +1.1 for Yb1 and +1.0 for Yb2, and display quasi-spherical shapes, typical for rare-earth and alkaline-earth cations in similar compounds.^54−56^

Their effective charges are quite low if compared with the formal values, suggesting an active participation in chemical bonding. Germanium and palladium are both QTAIM anions and display very similar shapes and charges. In fact, flat surfaces are found for Ge–Ge and Ge–Pd contacts whereas rather convex surfaces point toward the six-coordinating ytterbium atoms. Interestingly, these characteristic features of covalently bonded *p*-block elements are practically identical for both germanium and palladium. Such intriguing anionic behavior of palladium and other transition metals (e.g., Ru, Ir, Pt, Au, Ag) has been increasingly reported in the literature^54,55,57−63^ and considered responsible for unprecedented chemical properties.^64,65^ Hence, the QTAIM effective charges indicate that the crystal structure of Yb_2_PdGe_3_ is composed of  honeycomb anionic layers spaced by Yb cations.

To shed more light on the interactions among the constituents, a careful analysis of the topology of the Electron Localizability Indicator, in its ELI-D representation,^66,67^ was undertaken. Additionally, the crystal space is partitioned into valence and core basins by applying the Bader’s mathematical formalism to the ELI-D scalar field. The polarity of valence basins is evaluated through the intersection technique.^68^ The contribution, in terms of electronic population, of a QTAIM atom (*X*) intersecting an ELI-D basin (*B*_i_) is quantified by the bond fraction *p*(*B*_i_^*X*^).^69,70^ The latter are further used to evaluate the covalent, *cc*(*B*_i_), and lone pair, *lpc*(*B*_i_), characters^69,70^ for each ELI-D valence basin. These tools permit to describe ELI-D basins as lone pairs, nonpolar, and heteropolar bonds, opening the door to a consistent and quantitative treatment of the latter.

**Figure 9:**
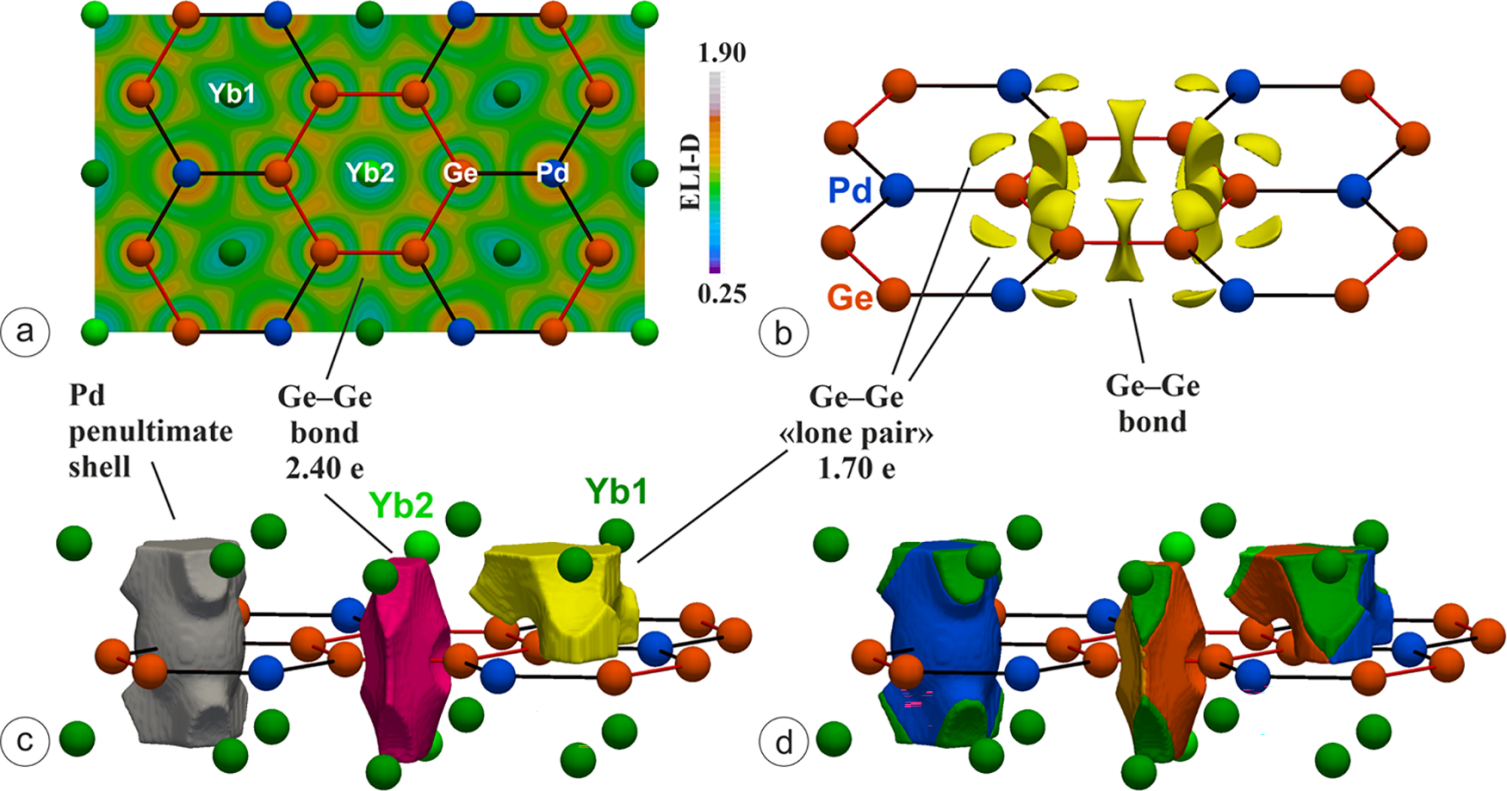
(a) ELI-D distribution within the (001) plane and (b) isosurfaces enclosing 1.176-localization domains; (c) shape of the ELI-D basins; (d) intersection of the ELI-D basins by QTAIM atoms; the coloring scheme for the intersected regions follows that chosen for the atoms and QTAIM basins. Average electronic populations of selected ELI-D basins are also indicated.

In the valence region, the ELI-D attractors are located around the germanium atoms, as shown in Figure 9a,b by means of its planar distribution and isosurfaces, respectively.

The presence of covalent Ge–Ge bonding is confirmed by the ELI-D maxima distribution. A tiny splitting of the attractors is observed for this interaction that does not substantially affect the overall bonding interpretation (more details on this are discussed in Figure S6). Two maxima per germanium atom point toward the neighboring metals and may be interpreted at a first approximation as lone pairs (Figure 9b). No attractors are found along Pd–Ge, so that a graphite-like bonding scenario is unlikely.

The described ELI-D topology composed of two lone pairs and two bonds per germanium is quite characteristic for two-bonded (2*b*) species and has been recently reported for both binary and ternary germanides within Ge zigzag chains (e.g., *RE*_2_*M*Ge_6_, CaGe,^58^ and LuGe^71^). Nonetheless, interesting differences may be detected when focusing on the average electronic populations. The bonding basins display a larger population than the lone pair ones, i.e., 2.40 vs 1.70 (see Figure 9c). This is in contrast with the expected overpopulation of lone pairs, along with a consequent bonds underpopulation, with respect to the ideal 2.00 electrons (e^–^).^58,71,72^ A comparative analysis with related intermetallic germanides is helpful; *RE*_2_*M*Ge_6_ (*M* = another metal) compounds are particularly suitable for this purpose due to similar Ge coordination environments (see Figure S7). In Y_2_PdGe_6_,^58^ the population of 1.68 e^–^, found in the bonding basin associated to Ge–Ge contacts at 2.45 Å, is significantly lower than the 2.40 e^–^ for the same basin in the title phase. In the analogous germanides, these populations were found to decrease together with increasing *d*_Ge–Ge_, reaching the value of 1.12 e^–^ for CaGe with a distance of 2.59 Å. Despite the structural similarities, Yb_2_PdGe_3_ is not following this trend and its enhanced bonding basin population can be considered an indication of a Ge–Ge bond order larger than one.

The bonding basin is intersected by two germanium and four ytterbium QTAIM atoms (see Figure 9d, orange and green portions). The contribution of the Yb species is negligible (0.12 e^–^ in total) so that it is an effectively two-atomic (2*a*) interaction. In accordance with its nonpolar nature, the *cc*(*B*_i_) is equal to 1. The valence basins indicated up to this point as “lone pairs” are intersected by 1Ge, 1Pd, 2Yb1, and 1Yb2. The main contribution comes from Ge, followed by Pd, leading to bond fraction values of 0.73 and 0.20, respectively, hinting toward a polar covalent bond rather than a lone pair. Whereas the Yb2 contribution should be neglected (0.012 e^–^), a different scenario is realized by the two Yb1 species. In fact, they yield a total bond fraction of 0.06 (0.03 per Yb2 atom), allowing to describe this basin as four-atomic (4*a*). Analogous bond fractions were recently reported for CaGe^58^ (0.08 per 4Ca atoms), where each Ge “lone pair” was definitely interpreted as a 5*a*-Ge_1_Ca_4_ basin after the application of the Penultimate Shell Correction method (PSC0), leading to a bond fraction of 0.15. Such approach was introduced to account for the rare-earth underestimated contributions due to a considerable charge storage in the penultimate shell. The same effect, although less severe, was reported for Ca, which displayed a core overpopulation of about 0.3 e^–^; for the actual calculations, a storage of 0.4 e^–^ was obtained for Yb. The PSC0 approach is still not applicable to compounds containing transition metals with an ambiguous oxidation state, such as Pd. The *cc*(*B*_i_) and *lpc*(*B*_i_) for such 4*a*-Ge_1_Pd_1_Yb1_2_ heteropolar bond are almost equal, being 0.53 and 0.47, respectively, supporting its covalent nature.

The shape of the Pd penultimate shell basin deserves further comments. It has six bulges that extend in the valence region (see Figure 9c) toward the six adjacent Yb1 species. In fact, each bulge is intersected by the corresponding Yb1 QTAIM atom (see dark green regions in Figure 9d). This kind of feature was observed for ternary intermetallics both with germanium, such as La_2_*M*Ge_6_ (*M* = Pd, Ag)^58^ and without germanium, such as LaAuMg_2_,^55^ and described as 2*a* polar covalent metal–metal bonds, as also confirmed by the ELI-D relative Laplacian. Considering this, each Pd is covalently bonded with the six neighboring Yb1 species, located on the vertices of the trigonal coordination prism. The realization of *RE*–Pd covalent interactions was also reported for some related ternary germanides on the basis of different quantum-chemical approaches.^54,73^

### 3.5 Additional Details on Chemical Bonding: From Molecules to Solid State

**Figure 10:**
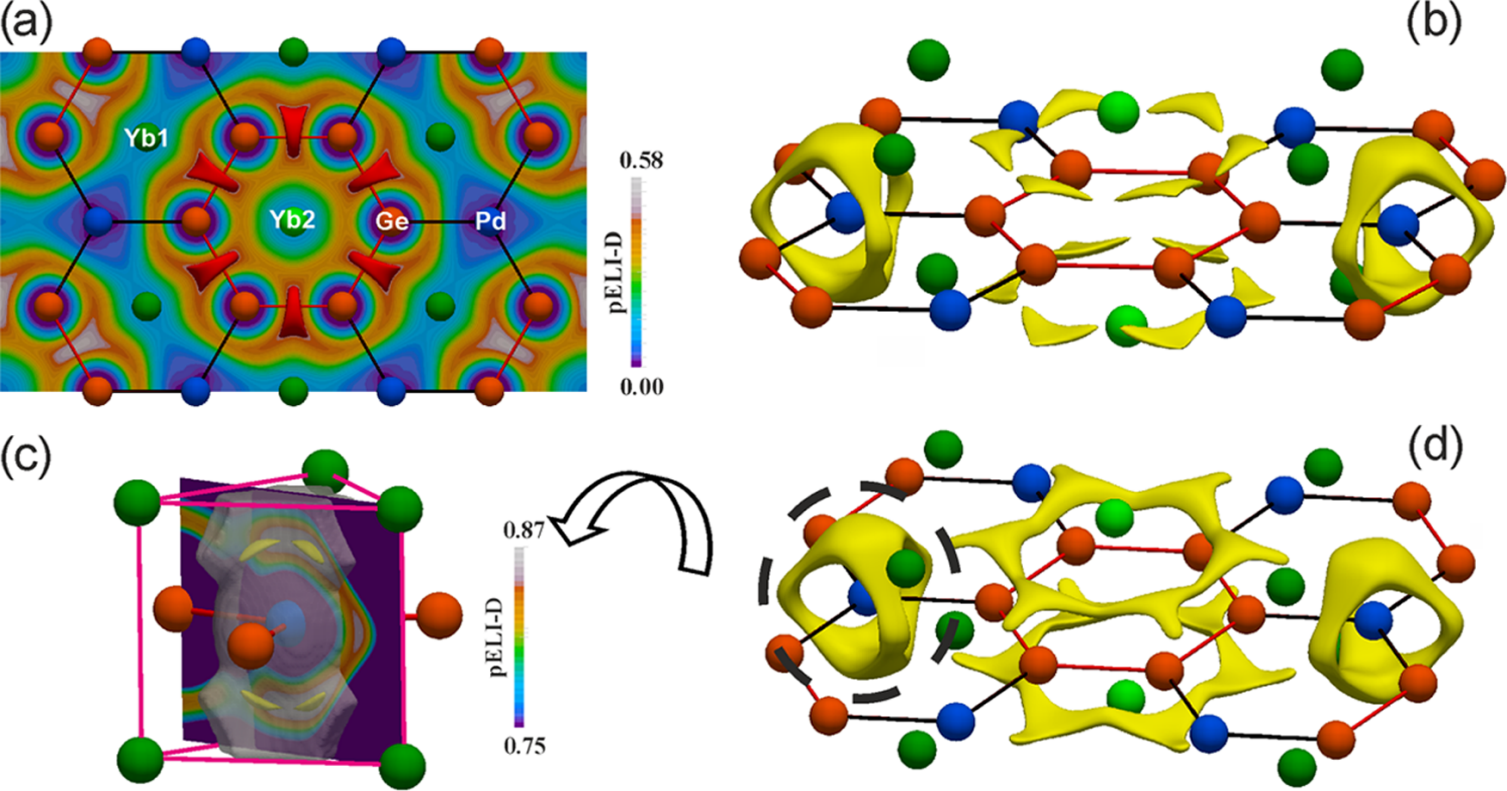
Partial ELI-D (pELI) contributions for A (−12.0 to −5.71 eV) and B (−5.71 to *E*_F_) DOS regions: (a) slice with pELI distribution derived from region A in the honeycomb layer together with red isosurfaces enclosing 0.46-localization domains; (b–d) yellow pELI isosurfaces derived from region B enclosing 0.833- (b), 0.867- (c), and 0.823-localization domains (d). The penultimate shell ELI-D basin of Pd is shown as well (c).

Further interesting details may be obtained analyzing the partial ELI-D (pELI, see Figure 10)^44,67^ that is calculated from the EDs obtained from separated states located in two DOS energy ranges (see Figure S8), indicated in the following with letters A (−12.0 eV < *E* < −5.71 eV) and B (−5.71 eV < *E* < 0.00 eV).

**Figure 11:**
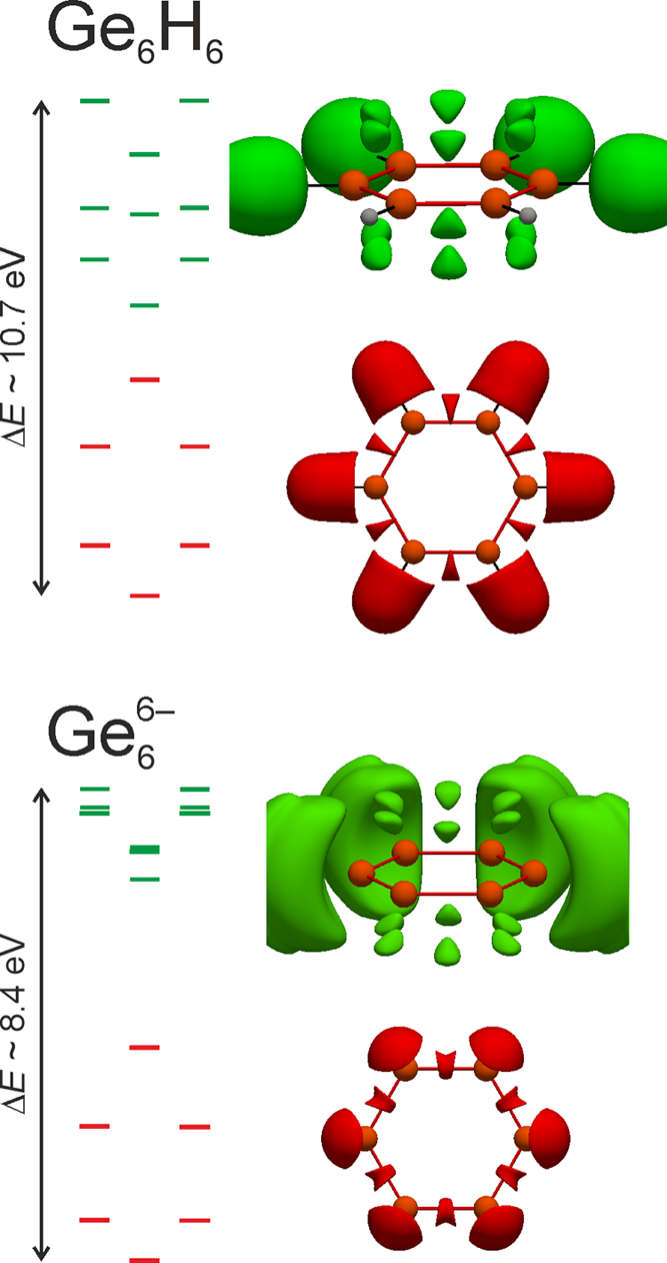
Molecular orbital energy diagrams of Ge_6_H_6_ (top) and Ge_6_^6–^ (bottom), together with pELI contributions. In the two figures representing pELI contributions derived from the highest energy orbitals (green), two domains in the exocyclic regions have been deleted to enable a better view for the reader.

Region A was selected since it is mainly dominated by the Ge 4*s* states and follows the same energy sequence found in the orbital schemes of Ge_6_H_6_ and Ge_6_^6–^ (red orbitals in Figure 11).

In fact, states in A are separated into four bands populated by 2, 4, 4, and 2 e^–^ per Ge_6_ ring of Yb_2_PdGe_3_. Thus, a comparative bonding analysis among the title crystalline solid and the molecules is enabled by the pELI function, which allows to recover orbital contributions in position space. Such comparison is even more interesting, keeping in mind that the two molecules both show one a_2u_ and two e_1g_ molecular orbitals with a shape characteristic for aromatic six-membered rings (see Figure S9). At this point, it is worth mentioning that based on computed magnetic criteria, it has been proven that Ge_6_H_6_, as its Si_6_H_6_ analogue, is less aromatic than benzene so that a nonplanar 3̅*m* (*D*_3d_) cyclic molecule is expected to be energetically favored.^74^ When the molecular structure optimization was performed starting from a planar 6/*mmm* molecule, it converges to a local energy minimum retaining the desired symmetry. On the contrary, if one Ge atom is located out of the plane, the final structure is the favored one being more stable by −0.563 eV (PW), −0.768 eV (PBE), and −0.784 eV (B3LYP) than 6/*mmm*, in accordance with the literature.^74^ Aiming at performing a consistent analysis, all-electron wave functions obtained from LDA/PW calculations were used for the comparative study. However, no relevant differences were detected among ELI and pELI calculated on the basis of the wave functions obtained employing the PBE or B3LYP functional.

Coming back to Yb_2_PdGe_3_, the states in the A energy interval of the DOS display pELI contribution only around the Ge_6_ ring on its very same plane (see pELI planar distribution in Figure 10a), with maxima located between the Ge–Ge contacts (red isosurfaces in Figure 10a). The pELI distribution resulting from the six energetically lower valence orbitals of Ge_6_H_6_ and Ge_6_^6–^ is analogue (red isosurfaces in Figure 11).

It is not straightforward to extend this analysis to the remaining energy range. In fact, the B region of the DOS is not separated into bands so that it is not trivial to compare it with the discrete orbitals of the reference molecules. Hence, clear differences are expected to occur between the pELI contributions coming from the B interval of the DOS and the remaining nine valence MOs of the molecules, indicated by green bars in Figure 11. They are formed by a linear combination of Ge 4*p* and H 1*s* for Ge_6_H_6_ and just by the Ge 4*p* for Ge_6_^6–^. The resulting pELI (see green isosurfaces in Figure 11) distributions are practically identical and show two kinds of attractors: the first are symmetrically located above and below the mid-point of the Ge–Ge bond, representing the π-interactions; the second are in the exocyclic region signifying Ge–H bonds in the case of Ge_6_H_6_ and Ge lone pairs for Ge_6_^6–^. As expected, a somewhat different picture is obtained for Yb_2_PdGe_3_. The pELI attractors are still symmetrically positioned above and below the Ge_6_ planes but find their location in correspondence of the Ge atoms and not among them (see yellow isosurfaces in Figure 10b). Thus, they seem to indicate the polar interactions between Ge and the surrounding metals. Nevertheless, the visualization of pELI isosurfaces at lower values reveals two reducible localization domains spreading over the whole Ge_6_ fragments above and below their plane, pointing out rather high pELI contributions also in these regions. Such feature supports the idea of some π-interactions among the Ge atoms, even if reduced with respect to the selected molecular references. Consequently, the bonding scenario appears to be intermediate between that of a typical 2c–2e Ge–Ge bond and an aromatic picture, well in line with the conclusions drawn from the interatomic distances and the ELI-D basin population analysis.

Finally, the pELI distribution from the DOS region B gives some insights into the Pd–Yb bonds as well. Six pELI maxima around each palladium point toward the neighboring Yb, as displayed by the 0.867-irreducible localization domains shown in Figure 10c. When such attractors are visualized superimposed with the Pd penultimate shell basins (gray transparent basin in Figure 10c), they are located in the spatial region corresponding to the bulges. This finding is not just supporting the formation of Pd–Yb bonds but constitutes one more evidence of the correct afore-given interpretation of the bulges of ELI-D penultimate shell basins.

## 4 Conclusions

Ternary rare-earth germanides of nominal composition *RE*_33.3_Pd_16.7_Ge_50.0_ were reinvestigated along the *RE* series (*RE* = Y, La–Nd, Sm, Gd–Er, Yb) aiming at checking for the existence of ordered structures. The existence of the *RE*(Pd_*x*_Ge_1–*x*_)_2_ disordered phases, crystallizing with the *hP*3-AlB_2_ structure, has been confirmed with all *RE*, but Yb. X-ray diffraction analyses both on powders and single crystals, indicate that the Yb_2_PdGe_3_ compound is a four-order superstructure of AlB_2_, crystallizing with the *hP*12-Ce_2_CoSi_3_ type of structure. Based on refined structural data and the calculated QTAIM effective charges, the crystal structure may be described as composed of two-dimensional  honeycomb anionic layers spaced by Yb cations. Both DFT/LSDA + *U* calculations and measured magnetic susceptibility as a function of temperature hint toward a divalent state of Yb. Moreover, Yb_2_PdGe_3_ displays a type-II superconducting behavior below the critical temperature of 4 K, a feature shared only with the disordered Y(Pd_*x*_Ge_1–*x*_)_2_ phase. Position-space chemical bonding analysis indicates, in addition to homopolar Ge–Ge bonds, polar four-atomic Ge_1_Pd_1_Yb_2_ and two-atomic Pd–Yb bonds. Further insights, with main regards to the nature of Ge–Ge interactions within regular Ge_6_ hexagons, were obtained by analyzing the partial ELI-D field that enabled a comparison between the crystalline Yb_2_PdGe_3_ and the hypothetic Ge_6_H_6_ and Ge_6_^6–^ molecules. Finally, a bond order larger than one is proposed for Ge–Ge bonds, suggesting a bonding scenario intermediate between that of a typical 2c–2e Ge–Ge bond and an aromatic behavior. Such results extend the chemistry of inorganic germanium and enrich with one more representative the bonding outcomes for ternary *RE*–Pd–Ge, which shows similar features like multiatomic interactions involving all species and *RE*–Pd bonds, here presented for the first time with *RE* = Yb. The search for appropriate doping, able to increase the measured critical temperature, and for new *RE*_2_*T*Ge_3_ representative, displaying intriguing physical and chemical properties, is ongoing.

## Supporting Information Available

Results of EDXS and XRPD analysis for *RE*–Pd–Ge samples, X-ray powder patterns for some representative samples, optimized structural data for the simulated Ge_6_H_6_ and Ge_6_^6–^ molecules, superconducting volume fraction, band structure and Brillouin zone for Yb_2_PdGe_3_, DOS and pDOS for Yb_2_PdGe_3_ and Ca_2_PdGe_3_ based on both DFT and DFT + *U* calculations, ELI-D attractors splitting along the Ge–Ge contacts, structural similarities among Yb_2_PdGe_3_ and Yb_2_PdGe_6_, DOS energy windows selected to calculate the pELI-D of Yb_2_PdGe_3_, and π molecular orbitals of the simulated Ge_6_H_6_ and Ge_6_^6–^ molecules (PDF)
