# Supplementary material for: Flux Growth, Crystal Structure, and Chemical Bonding of Yb2PdGe3, an AlB2 Superstructure within the Rare-Earth Series
Source: Inorg Chem. 2023 Jan 20;62(5):1988–99. doi: 10.1021/acs.inorgchem.2c03303 (PMC9906778; doi:10.1021/acs.inorgchem.2c03303)
Supplement: Supplementary file 1 — ic2c03303_si_001.pdf [file ic2c03303_si_001.pdf]

Supporting Information

Flux growth, crystal structure and chemical bonding of Yb_2PdGe_3, an AlB_2 superstructure within the rare earth series

Riccardo Freccero[1*], Laura C. J. Pereira[2], Pavlo Solokha[1], Serena De Negri[1]

[1]*Università degli Studi di Genova, Dipartimento di Chimica e Chimica Industriale, Via Dodecaneso 31, I-16146 Genova, Italy*

[2]*Centro de Ciências e Tecnologias Nucleares, Department of Engenharia e Ciências Nucleares, Instituto Superior Técnico, Universidade Lisboa, Estrada Nacional 10, 2695-066 Bobadela, Portugal*

* To whom the correspondence should be addressed: Riccardo Freccero; E-mail: riccardo.freccero@.unige.it

Table S1. SEM/EDXS (at.%) and XRD data on the investigated *RE*-Pd-Ge samples (annealed at 800°C for 3 weeks). When the Pearson symbol is not reported the number of peaks in the powder diffraction pattern was not sufficient to unambiguously assign it. Asterisks (*) indicate samples containing unknown phases.

RE	**Global EDXS Composition [at%]**			**Phases**	**EDXS Composition [at%]**			**Crystal Structure**	**Lattice Parameters [Å]**	
	RE	**Pd**	**Ge**		***RE***	**Pd**	**Ge**		***a***	***c***
**Y***	34.1	16.8	49.1	$Y(Pd_xGe_{1-x})_2$<br>$YPdGe_2$	34.8<br>23.5	17.7<br>22.6	47.5<br>53.9	*hP*3-AlB_2<br>*oI*32-$YIrGe_2$	4.186(1)	4.000(1)
**La**	33.4	17.2	49.4	$La(Pd_xGe_{1-x})_2$<br>$LaPdGe_2$	33.5<br>25.5	17.1<br>24.8	49.4<br>49.7	*tI*12-$ThSi_2$<br>*oS*16-$CeNiSi_2$	4.293(1)	14.891(4)
**Ce**	33.6	17.5	48.9	$Ce(Pd_xGe_{1-x})_2$<br>$Ce_2Pd_3Ge_5$	34.1<br>21.4	17.4<br>30.3	48.5<br>48.3	*tP*12-$CePd_{0.5}Ge_{1.5}$	4.247(2)	14.801(7)
**Pr**	31.1	18.5	50.4	$Pr(Pd_xGe_{1-x})_2$<br>$Pr_2Pd_3Ge_5$	31.7<br>18.5	17.8<br>32.1	50.6<br>49.4	*hP*3-AlB_2<br>*oI*40-$U_2Co_3Si_5$	4.245(1)	4.219(2)
**Nd**	32.8	17.7	49.8	$Nd(Pd_xGe_{1-x})_2$<br>$Nd_2Pd_3Ge_5$	32.6<br>19.7	17.0<br>30.8	50.4<br>49.5	*hP*3-AlB_2<br>*oI*40-$U_2Co_3Si_5$	4.233(1)	4.185(1)
**Sm**	30.9	17.5	51.6	$Sm(Pd_xGe_{1-x})_2$<br>$SmPdGe_2$<br>Sm_2PdGe_6	33.4<br>24.1<br>21.9	17.5<br>26.8<br>12.1	49.1<br>49.1<br>66.0	*hP*3-AlB_2<br>*oI*40-$U_2Co_3Si_5$<br>*oS*18-Ce_2CuGe_6	4.212(1)	4.117(1)
**Gd***	32.9	16.8	50.3	$Gd(Pd_xGe_{1-x})_2$<br>Gd_2PdGe_6	33.6<br>22.9	18.8<br>12.4	47.6<br>64.7	*hP*3-AlB_2	4.207(1)	4.052(1)
**Tb***	33.1	17.3	49.6	$Tb(Pd_xGe_{1-x})_2$	33.0	20.5	46.5	*hP*3-AlB_2	4.216(1)	3.981(1)
**Dy***	32.2	18.1	49.7	$Dy(Pd_xGe_{1-x})_2$<br>Dy_2PdGe_6	33.1<br>23.1	22.0<br>12.4	44.9<br>64.5	*hP*3-AlB_2	4.229(1)	3.906(1)
**Ho***	37.6	17.6	44.8	$Ho(Pd_xGe_{1-x})_2$	34.2	22.6	43.2	*hP*3-AlB_2	4.231(1)	3.851(1)
**Er***	35.7	9.8	54.5	$Er(Pd_xGe_{1-x})_2$	31.3	21.5	47.2	*hP*3-AlB_2	4.239(1)	3.789(1)
**Yb**	33.6	18.4	48.0	Yb_2PdGe_3	34.6	16.9	48.5	*hP*12-Ce_2CoSi_3	8.463(1)	4.073(1)

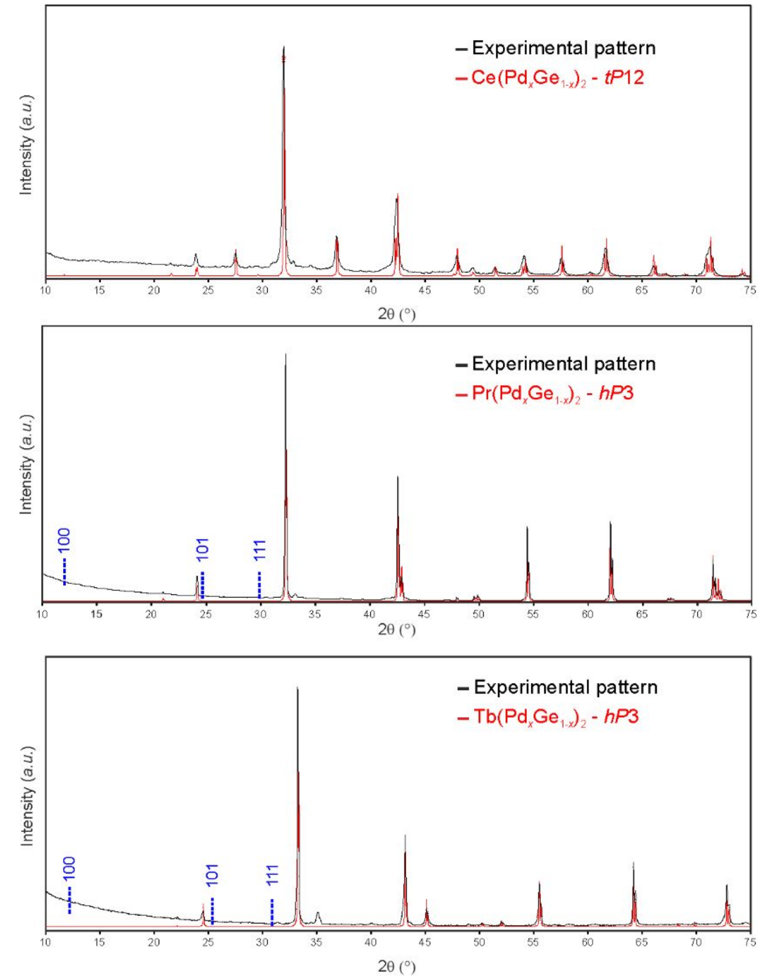

Figure S1. X-ray powder patterns for the Ce-Pd-Ge, Pr-Pd-Ge, and Tb-Pd-Ge samples selected as representatives for light and heavy *RE*. Blue dashed lines for *RE* = Pr, Tb point out both the positions and Miller indexes of the missing super-reflections for the hypothetic RE_2PdGe_3 ordered compound.

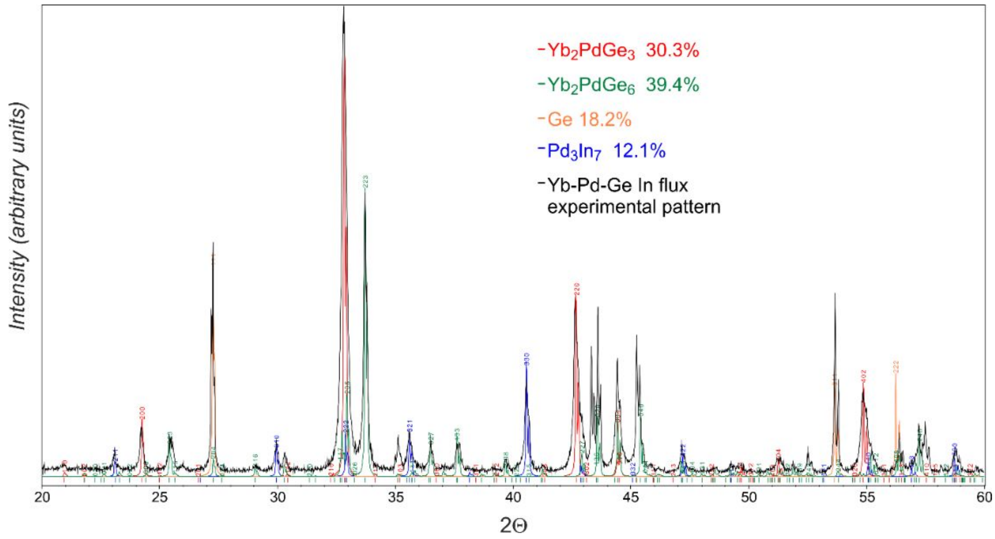

Figure S2. X-ray powder pattern for the Yb-Pd-Ge sample synthesized in metal flux after In removal

Table S2. Interatomic distances for Ge_6H_6 and Ge_6^{6-} optimized employing different exchange-correlation functional (f_{xc}): the Perdew and Wang (PW) for the local-density approximation (LDA), the Perdew, Burke and Ernzerhof (PBE) for the generalized-gradient approximation (GGA), and the B3LYP hybrid functional.

Compound	f_{xc}	$d_{Ge–Ge}$ [Å]	$d_{Ge–H}$ [Å]
Ge_6He_6	pw	2.26	1.53
	pbe	2.30	1.53
	b3lyp	2.29	1.52
Ge_6^{6-}	pw	2.68	/
	pbe	2.77	
	b3lyp	2.77	

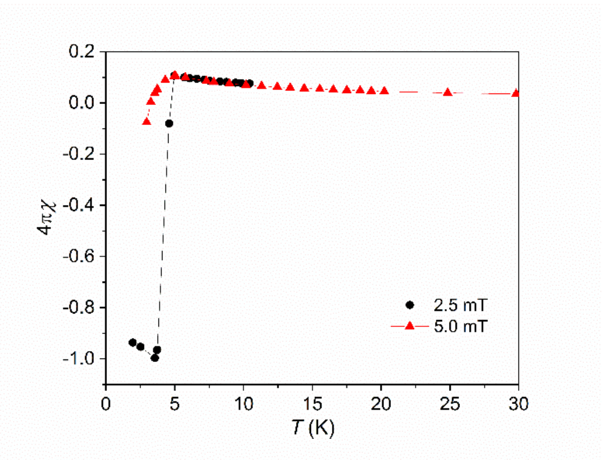

Figure S3. Superconducting volume fraction of Yb_2PdGe_3 at 2.5 and 5.0 mT.

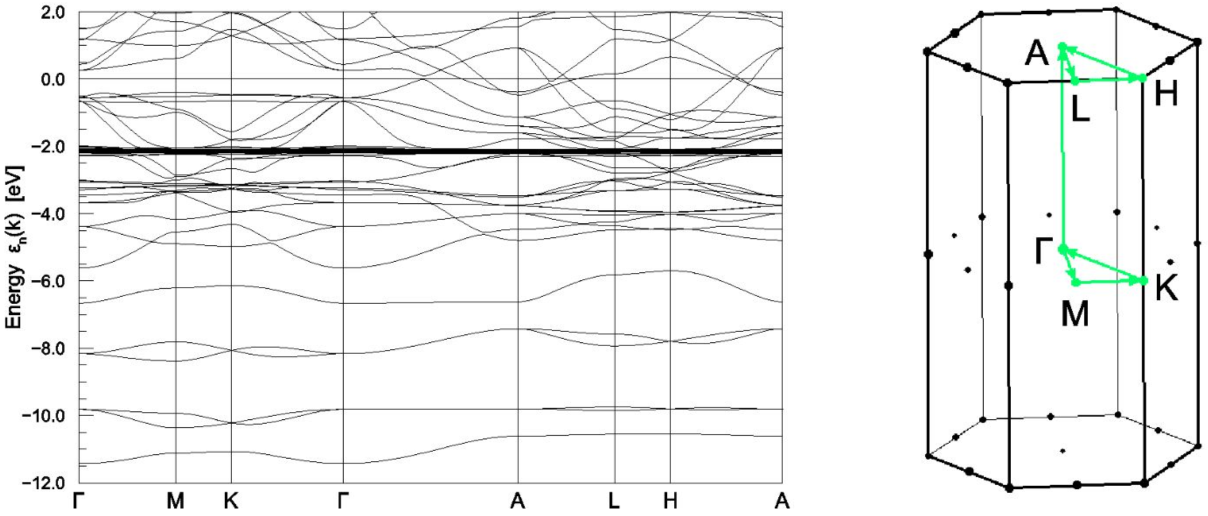

Figure S4. Band structure and first Brillouin zone for Yb_2PdGe_3. The selected k-path, indicated by green arrows, and the Γ (0, 0, 0), M ($\frac{1}{2}$, 0, 0), K ($\frac{2}{3}$, $\frac{1}{3}$, 0), A (0, 0, $\frac{1}{2}$), L ($\frac{1}{2}$, 0, $\frac{1}{2}$) and H ($\frac{2}{3}$, $\frac{1}{3}$, $\frac{1}{2}$) symmetry points and lines are shown into the Brillouin zone.

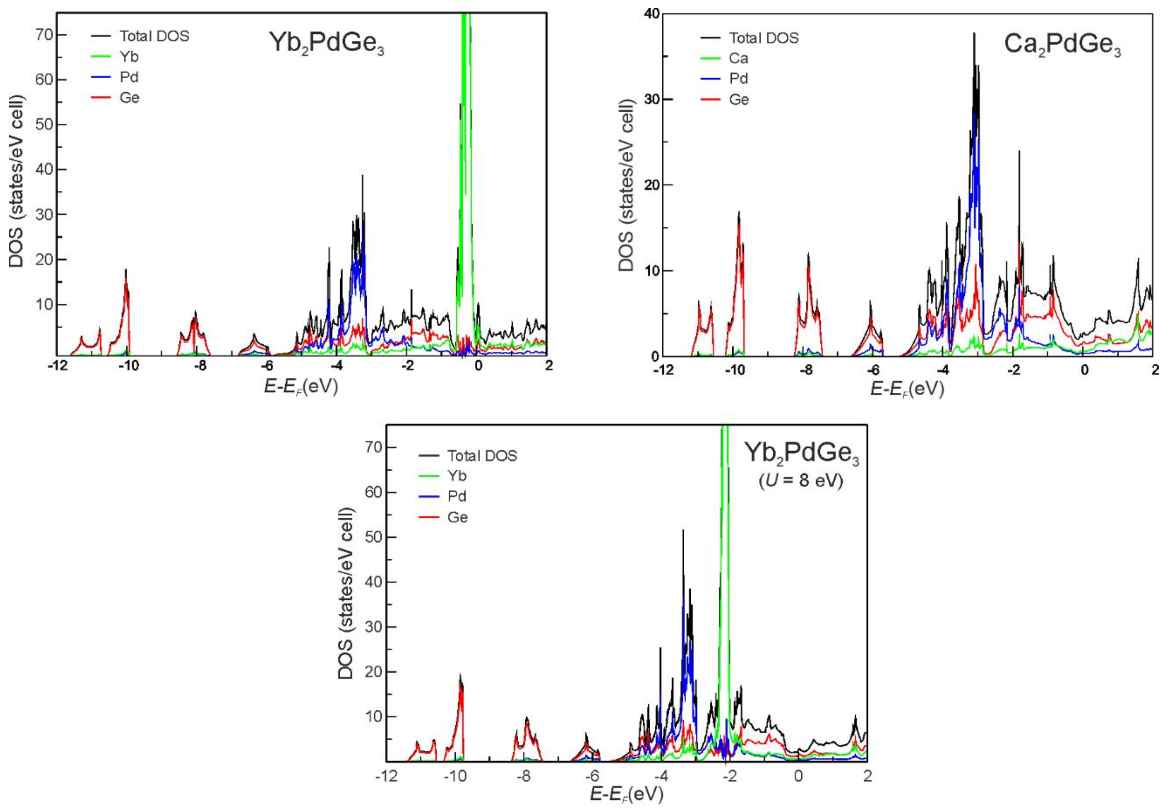

Figure S5. Total and atom-projected Density of States (DOS/*p*DOS) for Yb_2PdGe_3 (top left) and Ca_2PdGe_3 (top right) calculated at a DFT/LSDA (PW) level of theory. The 4*f* states of the Yb species are located close to E_F, as expected when a Hubbard U parameter is not employed. To enable a better comparison among the DOS curves, that for Yb_2PdGe_3 generated with a DFT + U treatment is also shown (bottom).

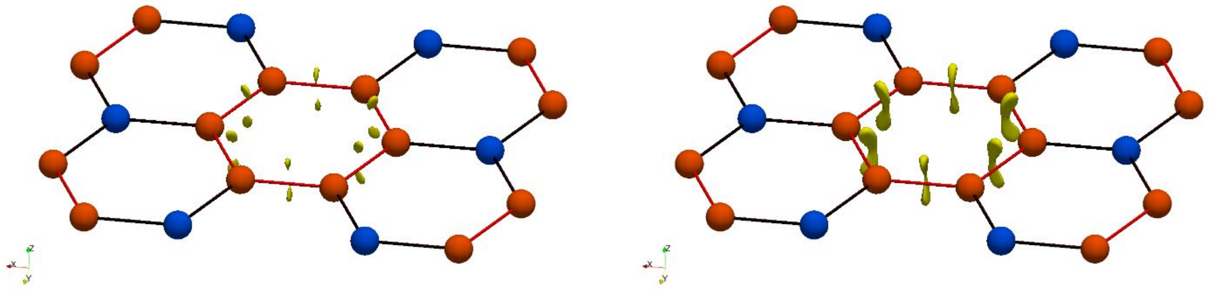

Figure S6. Isosurfaces generated with ELI-D values of 1.215 (left) and 1.210 (right).

A splitting of the ELI-D attractors occurs for Ge–Ge interactions, so that there are two instead of one along the germanium homocontacts, being symmetrically located above and below them with respect to the honeycomb Ge/Pd layer (Figure S6, left). They merge into one reducible localization domain (Figure S6, right) at a slightly lower ELI-D value, i.e. 0.01, than at the attractor. A similar feature was described for the calculated ELF of the $SrGa_2$ and $BaGa_2$ gallides[1] along the Ga–Ga contacts. Interestingly, the same does not hold for metal diborides[2] nor for benzene[3], probably suggesting that

this splitting is more prone to occur moving down along the periodic table groups. Since this very tiny effect does not affect the overall bonding interpretation, one basin set was generated for each Ge–Ge bonding (purple basin in Figure 9c).

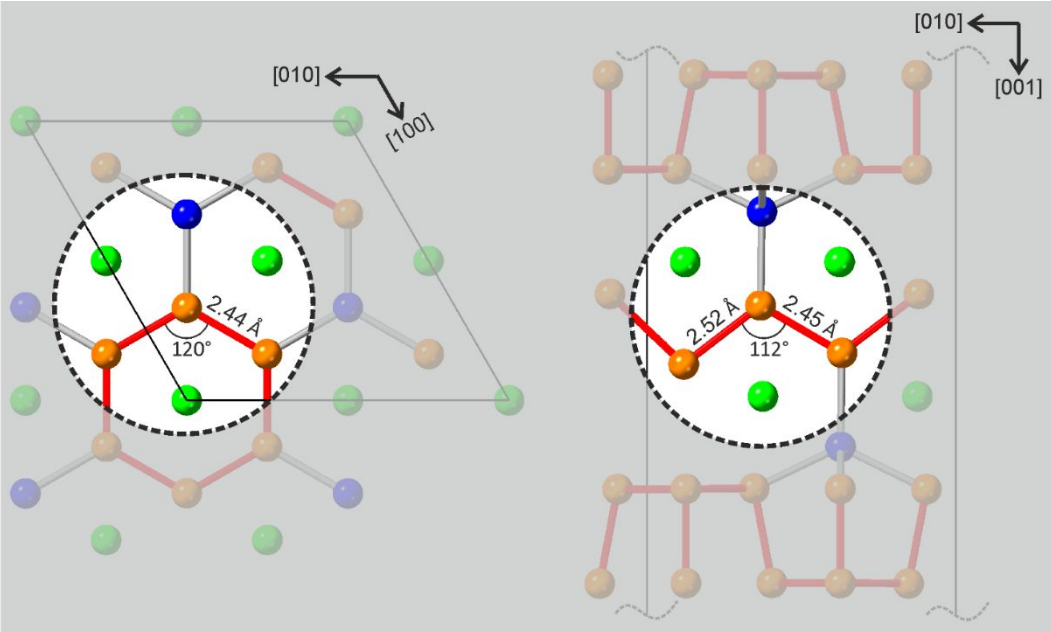

Figure S7. Structural similarities among the coordination environments of Ge species within Yb_2PdGe_3 and Y_2PdGe_6.

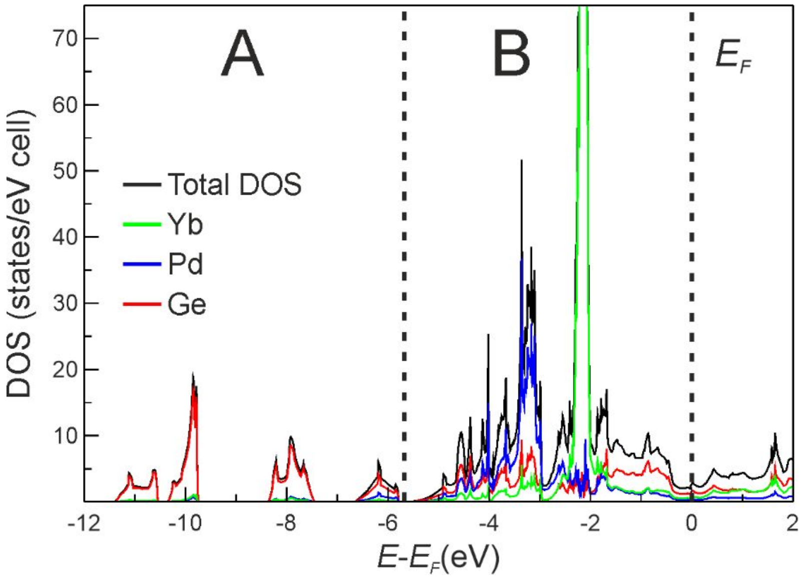

Figure S8. DOS and *p*DOS for Yb_2PdGe_3 (DFT+*U*). The A and B regions selected for the partial ELI-D (pELI) calculation are highlighted.

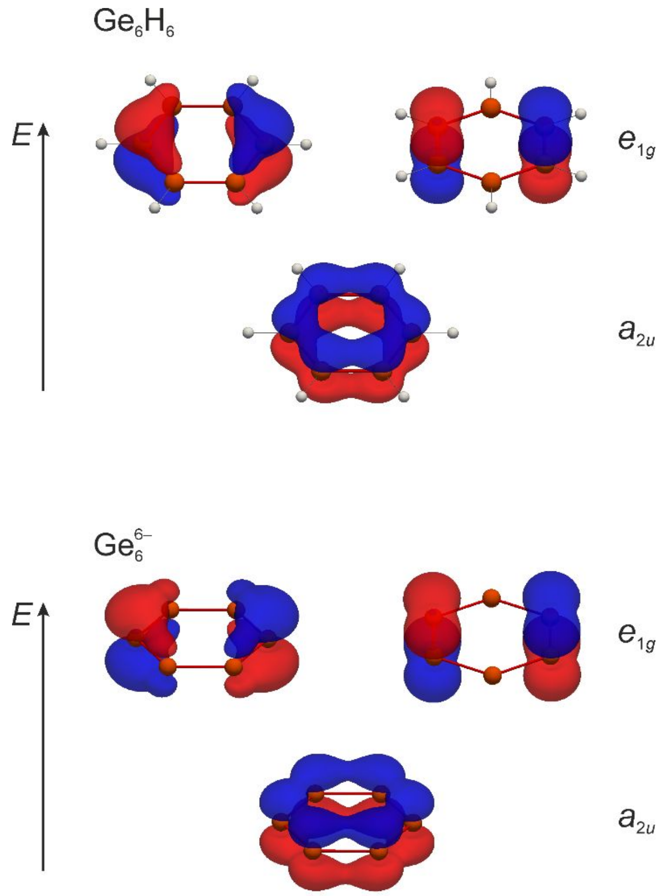

Figure S9. The a_{2u} and e_{1g} π Kohn-Sham orbitals for the Ge_6H_6 and Ge_6^{6-} molecules. Orbitals are represented by means of isosurfaces with absolute values of 0.040 and 0.030 e/bohr^3 for Ge_6H_6 and Ge_6^{6-}, respectively. Blue and red denote positive and negative values, respectively.
